# Van der Waals interfacial reconstruction in monolayer transition-metal dichalcogenides and gold heterojunctions

**DOI:** 10.1038/s41467-020-14753-8

**Published:** 2020-02-21

**Authors:** Ruichun Luo, Wen Wu Xu, Yongzheng Zhang, Ziqian Wang, Xiaodong Wang, Yi Gao, Pan Liu, Mingwei Chen

**Affiliations:** 1 Shanghai Key Laboratory of Advanced High-temperature Materials and Precision Forming, State Key Laboratory of Metal Matrix Composites, School of Materials Science and Engineering, Shanghai Jiao Tong University, Shanghai, 200240 P. R. China; 2 Department of Materials Science and Engineering, Johns Hopkins University, Baltimore, MD 21218 USA; 3 Department of Physics, School of Physical Science and Technology, Ningbo University, Ningbo, 315211 P. R. China; 4 WPI Advanced Institute for Materials Research, Tohoku University, Sendai, 980-8577 Japan; 5 Shanghai Advanced Research Institute, Chinese Academy of Sciences, Shanghai, 201210 P. R. China

**Keywords:** Materials science, Two-dimensional materials

## Abstract

The structures and properties of van der Waals (vdW) heterojunctions between semiconducting two-dimensional transition-metal dichalcogenides (2D TMDs) and conductive metals, such as gold, significantly influence the performances of 2D-TMD based electronic devices. Chemical vapor deposition is one of the most promising approaches for large-scale synthesis and fabrication of 2D TMD electronics with naturally formed TMD/metal vdW interfaces. However, the structure and chemistry of the vdW interfaces are less known. Here we report the interfacial reconstruction between TMD monolayers and gold substrates. The participation of sulfur leads to the reconstruction of Au {001} surface with the formation of a metastable Au_4_S_4_ interfacial phase which is stabilized by the top MoS_2_ and WS_2_ monolayers. Moreover, the enhanced vdW interaction between the reconstructed Au_4_S_4_ interfacial phase and TMD monolayers results in the transition from n-type TMD-Au Schottky contact to p-type one with reduced energy barrier height.

## Abstract (web summary)

Material interfaces are highly crucial to improve device performances. Here, the authors report interfacial reconstruction at the Au {001} substrate surface and the top MoS_2_ and WS_2_ monolayers due to the formation of a metastable interfacial Au_4_S_4_ phase. The reconstructed interface results in a p-type Schottky contact with reduced barrier height.

## Introduction

Semiconducting 2D TMDs, such as MoS_2_ and WS_2_, have shown exceptional electronic^1^, optical^2^, and catalytic^3^ properties superior to their bulk counterparts. To employ 2D TMDs in electronic devices, one of the essential processes is to connect the atomically-thick crystals with bulk metals that serve as electrodes, connectors and supporting substrates^4–6^. The structure and chemistry of 2D semiconductor-metal interfaces significantly influence the performances of 2D electronic devices^7^. Two types of contacts, covalent and vdW interfaces from Ti-MoS_2_ and Au-MoS_2_, have been suggested by previous theoretical invetigations^8,9^. However, it has been found that both interfaces encounter the fermi-level pinning^10^ and, thus, performance degradation of the heterostructures that are fabricated by depositing or lithographing metals onto 2D TMDs^11,12^. Atomic scale characterization of the interfaces by scanning transmission electron microscopy (STEM) has suggested that the degradation is associated with the damage of TMDs in the contact layer by invasion of metal atoms^12–14^. Interface engineering, such as insertion of additional vdW layers (graphene^15^, hexagonal boron nitride (hBN)^16^, etc.), mechanical transfer of metal films^13^ and incorporation with indium^17^, are explored to form ideal vdW contacts that are free from the chemical disorder.

On the other hand, chemical vapor deposition (CVD) is a well-developed method to grow high quality 2D materials and to fabricate electronic devices on various substrates^18^, which provides the possibility to directly obtain perfect vdW contacts with metals, like graphene grown on Cu (111) surface^19^ and hBN grown on Cu (110) surface^20^. As to the CVD growth of TMDs, insulating substrates are commonly used^21^ and Au is almost the only metal substrate and is known to form vdW interfaces with TMDs thanks to its chemical stability in chalcogen-rich environments during CVD^22–25^. Although Au is inert in bulk form, Au surface atoms has rich ligand chemistry partially due to the existence of possible oxidation states of gold. Various Au-thiolate complexes and low-temperature chemisorbed sulfur have been observed on Au surfaces^26,27^. Accordingly, the vapored sulfur and S-Mo precursors formed during CVD reactions may affect the atomic structures of gold surfaces and, subsequently, alter interfacial interactions with as-deposited 2D TMDs. Experimentally, enhanced electrocatalytic activities and quenched photoluminescence from possible electron coupling have been observed from monolayer MoS_2_ grown on gold substrates^22,23,28^. In addition, Au substrates are found to arouse the 1H-1T phase transformation of MoS_2_ monolayers by inducing the in-plane shifting of S or/and Mo atom layers^22,29,30^. The interaction between monolayer MoS_2_ and gold substrates has been investigated by scanning tunneling microscope (STM) and periodically striped and moiré superstructures, most likely rising from the large lattice mismatch between the basal plane of MoS_2_ and Au (001) or (111)^31,32^, have been observed from the planar view. In contrast, the cross-sectional views, which can directly present the structure and chemistry of interfaces, have not been realized in the CVD grown TMD/Au junctions.

In this study, we report atomic-scale observations of the interfaces between Au and monolayer MoS_2_ and WS_2_ from cross-sectional views by growing the monolayer TMDs on 3D nanoporous gold (NPG). NPG has been demonstrated as an effective 3D substrate for CVD growth of TMDs^22,28^. The large surface area and high conductivity of NPG can dramatically enhance the electrocatalysis and photoelectrocatalysis of 2D TMDs. More importantly, plentiful facets with different crystallographic orientations of NPG offer a unique opportunity to directly observe the cross-sectional structures of the monolayer MoS_2_ on different surfaces of Au without additional TEM sample preparation, such as focused ion beam (FIB) milling and ion milling which may introduce unexpected interface damage. The pristine TMD/Au interfaces well preserve the structural integrity and show reconstructed interfaces with the formation of an unconventional Au_4_S_4_ superstructure on the topmost surface of Au {100}. Moreover, the enhanced vdW interaction between MoS_2_ and reconstructed Au surfaces leads to the transition from n-type TMD-Au Schottky contact to p-type one with reduced barrier height.

## Results

### Synthesis and characterization of monolayer TMDs on NPG

**Fig. 1:**
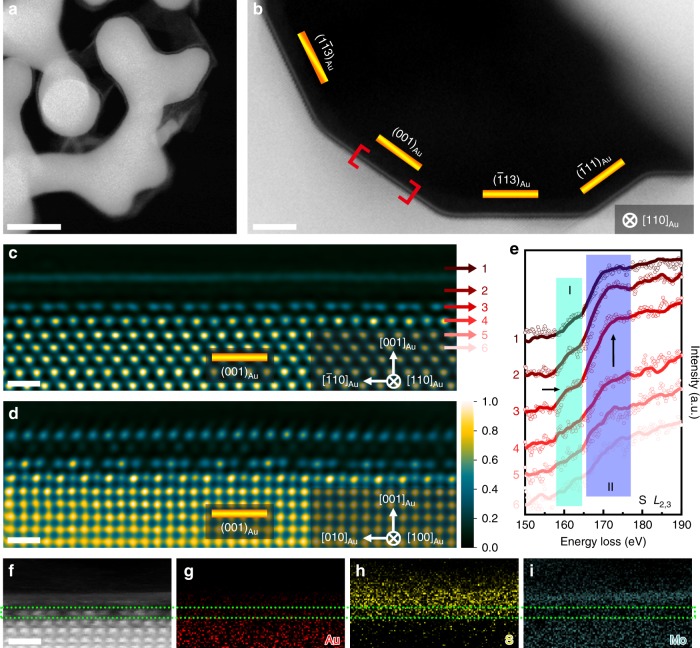
Structural and chemical characterization of the interface between monolayer MoS_2_ and NPG. **a** Low magnification HAADF-STEM image of the MoS_2_ film grown on NPG. Scale bar, 50 nm. **b** Magnified HAADF-STEM image of monolayer MoS_2_ grown on the internal surfaces of NPG. Scale bar, 5 nm. **c**, **d** Filtered HAADF-STEM images viewed from the [110]_Au_ (**c**) and [100]_Au_ (**d**) directions. Color variation from black to blue and then to yellow corresponds to the intensity from low to high. Scale bar, 0.5 nm. **e** EELS spectra of S *L*_2,3_ core-level edges summed from the different layers of MoS_2_ and Au in (**c**). **f**–**i** EELS elemental mappings of Au (**g**), S (**h**) and Mo (**i**) and simultaneous HAADF-STEM image (**f**) of monolayer MoS_2_ on Au (001) surface. Scale bar, 0.5 nm.

**Fig. 2:**
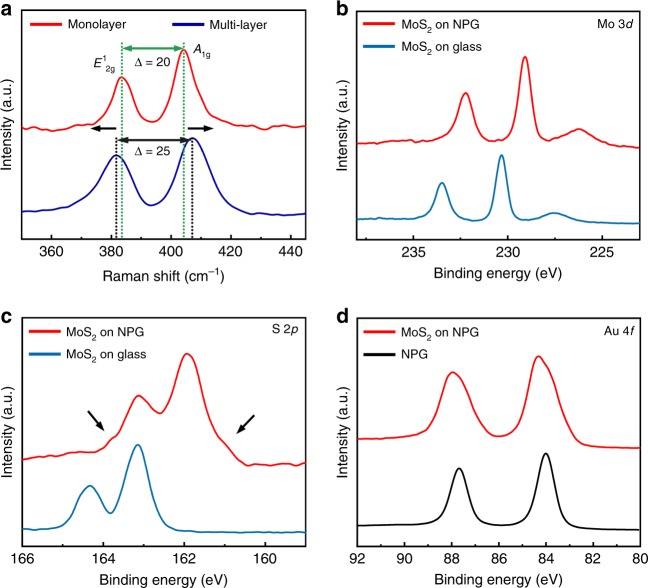
Raman and XPS characterizations of MoS_2_ growing on NPG. **a** Raman spectra of monolayer and multi-layer MoS_2_ on NPG. **b** Mo 3*d* and **c** S 2*p* core-level peaks of MoS_2_ grown on NPG and glass. **d** Au 4 *f* core-level peaks of original NPG and MoS_2_ grown on NPG.

MoS_2_ was grown on a 100 nm thick NPG film, which was prepared by chemical dealloying^33^, by a low-pressure CVD method (Supplementary Fig. 1)^34^. The high CVD temperature of 923 K leads to the slight coarsening of nanoporous structure of NPG from the as-dealloyed pore size of ~20 to ~50 nm (Supplementary Fig. 2). Meanwhile, the coarsening also leads to the development of multiple low-energy surface facets^35^ for cross-sectional TEM observations. Figure 1a shows the high angle annual dark-field (HAADF) STEM image of NPG covered by a thin layer of MoS_2_. By carefully optimizing the CVD conditions, monolayer MoS_2_ can be obtained, which is evidenced by STEM images (Fig. 1b), as well as Raman spectra (Fig. 2a) with a narrowed frequency difference (Δ ~ 20 cm^−1^ compared to the multi-layered value of ~25 cm^−1^) between *E*^1^_2g_ (~384 cm^−1^) and *A*_1g_ (~404 cm^−1^)^36^. X-ray photoemission spectroscopy (XPS) measurements (Fig. 2b, c) were also performed to characterize the formation of MoS_2_. The XPS spectra show the Mo 3*d*_5/2_ (229.1 eV) and Mo 3*d*_3/2_ (232.2 eV) peaks from Mo^4+^ as well as the S 2*p*_3/2_ (161.9 eV) and S 2*p*_1/2_ (163.1 eV) peaks from S^2−^ (ref. ^23^), indicating the formation of semiconducting 2D MoS_2_ (2H phase for multilayer and/or 1H phase for monolayer). By comparing with the XPS results of MoS_2_ grown on the glass substrate, large energy shifts of the Mo 3*d* and S 2*p* peaks of MoS_2_ on NPG can be seen. For Mo 3*d*_5/2_ and Mo 3*d*_3/2_ core levels, the peaks shift from 230.3 and 233.4 eV of MoS_2_ on glass to 229.1 and 232.2 eV of MoS_2_ on NPG. For S 2*p*_3/2_ and S 2*p*_1/2_ core levels, the binding energies shift from 163.1 and 164.3 eV of MoS_2_ on glass to 161.9 and 163.1 eV of MoS_2_ on NPG. The obvious binding energy changes imply an enhanced interaction between MoS_2_ and Au in comparison with MoS_2_ and glass. It is worth noting that the S 2*p* doublet peaks of MoS_2_ on NPG become broad and exhibit weak shoulders, marked by arrows, compared to those of MoS_2_ on glass. Correspondingly, the doublet peaks of Au 4*f* from NPG also become broad and asymmetric with a slightly blue shift of 0.3 eV after the growth of MoS_2_ (Fig. 2d), indicating the possible formation of Au–S bonding in the MoS_2_ on NPG system. Moreover, we performed the quantitative XPS analysis of the composition of MoS_2_ grown on glass and NPG. The corresponding quantifications of Mo and S in atomic % and their ratios from three independent measurements are shown in Supplementary Table 1. For the samples of MoS_2_ on glass, the Mo/S ratios are all larger than 0.5, the stoichiometric ratio of MoS_2_, which is consistent with previous reports that CVD grown MoS_2_ contains sulfur vacancies^37–39^. However, for the samples of MoS_2_ on NPG, the Mo/S ratios are all less than 0.5, suggesting the existence of excess sulfur in the system. Combined with the facts that the covalent bond between gold and sulfur could form^26,27^, the XPS results suggest the possible formation of the Au–S bonding after MoS_2_ growing on NPG.

**Fig. 3:**
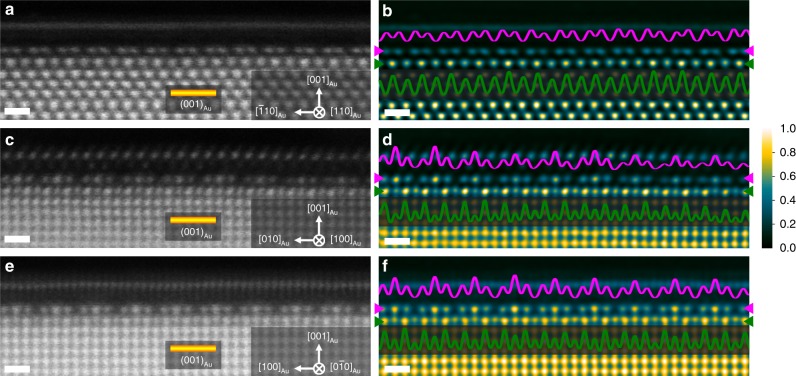
Structural characterizations of the interfaces between MoS_2_ monolayer and Au (001) surface of NPG. **a**, **c**, **e** Raw HAADF-STEM images viewed from the [110]_Au_ (**a**), [100]_Au_ (**c**) and [0$\bar 1$0] _Au_ (**e**) directions. Scale bar, 0.5 nm. **b**, **d**, **f** Filtered atomic-resolution HAADF-STEM images with false color viewed from the [110]_Au_ (**b**), [100]_Au_ (**c**) and [0$\bar 1$0] _Au_ (**e**) directions. Color variation from black to blue and then to yellow corresponds to the intensity from low to high. The magenta and green lines are intensity profiles measured along the Au topmost layer and sublayer following the corresponding arrows in (**b**, **d**) and (**f**), respectively. Scale bar, 0.5 nm.

The multiple terminal surfaces with different facets of NPG provide various Au-TMDs interfaces for direct cross-sectional observations^40,41^. A high resolution HAADF-STEM image (Fig. 1b) viewed along the Au [110] ([110]_Au_) direction shows an intact MoS_2_ monolayer attached on the gold surface with different facets, indicating the uniform growth of MoS_2_ on the internal surface of NPG. The zoom-in images of Au (001) surface along both the [110]_Au_ and [100]_Au_ directions (Fig. 1c, d) reveal the atomically sharp Au-MoS_2_ contact as well as the arrangements of S atoms in the semiconducting 1H MoS_2_ monolayer. Interestingly, an unusual periodic structure, different from the face-centered cubic (FCC) order of Au and 1H of MoS_2_, can be observed from the Au (001) surface underneath the monolayer MoS_2_ (Fig. 1c, d), indicating the occurrence of interfacial reconstruction during MoS_2_ growth. From the [110]_Au_ imaging direction (Fig. 1c), the periodic structure of the topmost Au (001) layer consists of paired atom columns with a lower intensity compared to the FCC matrix. Moreover, the visible intensity variation in a periodic manner can also be observed from the sublayer (the second Au atom layer counting from the topmost surface layer of Au) although the projected atomic occupations from the sublayer columns still locate at the FCC lattice. Since the intensity of each column is proportional to the Au atom number in the column, the periodic intensity variation indicates a regular change of Au atom numbers in the sublayer and thus a different ordering sequence. Consequently, the top two layers of the Au (001) surface experience a structural reconstruction during the CVD growth. From [100]_Au_ imaging direction (Fig. 1d), the interfacial reconstruction can be directly visualized as the topmost surface of Au (001) shows a trios structure in which the center atom columns have a contrast close to the FCC Au matrix and two side atom columns have a relatively weaker contrast. Meanwhile, the sublayer atom columns, viewed along [100]_Au_ direction, also show the reconstructed periodic intensity variation which is similar to that viewed from [110]_Au_ direction. The intensity line profiles of these two reconstructed Au layers, displaying the periodic ordering and intensity variation, can be seen in Fig. 3, together with the raw HAADF-STEM images. To the best of our knowledge, the distinctive structural periodicity at the interface between Au (001) and MoS_2_ monolayer has not been observed from reconstructed Au (001) surfaces before. Importantly, the same interfacial reconstruction can be observed from all the Au (001) facets covered by 1H MoS_2_ in this study (Supplementary Fig. 3), demonstrating the universality of the Au (001) surface reconstruction under 1H MoS_2_.

The chemistry of the reconstructed Au (001) surface under MoS_2_ was investigated by electron energy loss spectroscopy (EELS) with an atomic-scale spatial resolution under STEM mode. Figure 1e is the summed EELS spectra of S *L*_2,3_ core-level edges acquired from MoS_2_ to Au by layer-by-layer analysis. The dominating features of the S *L*_2,3_ fine structure in reign I and II is composed of S 3s and 3d partial density of states (DOS)^42^. The reconstructed topmost Au layer (Layer #3) shows a strong S signal, which is distinct from the lower layers (#4, #5, and #6) and suggests that sulfur is involved into the topmost surface reconstruction. In addition, the atomic-scale EELS measurements also reveal the elemental distributions of Au, Mo and S in the Au-MoS_2_ interface with monolayer MoS_2_ grown on Au (001) surface (Fig. 1g–i, Supplementary Fig. 4), together with simultaneous HAADF-STEM imaging (Fig. 1f), further confirming that the reconstructed Au (001) topmost surface is constituted by Au and S elements. However, it appears that sulfur does not invade into the sublayer of Au (001) surface since the EELS spectrum of Layer #4 is similar to Layer #5 and #6 (Fig. 1e) where S cannot be detected.

**Fig. 4:**
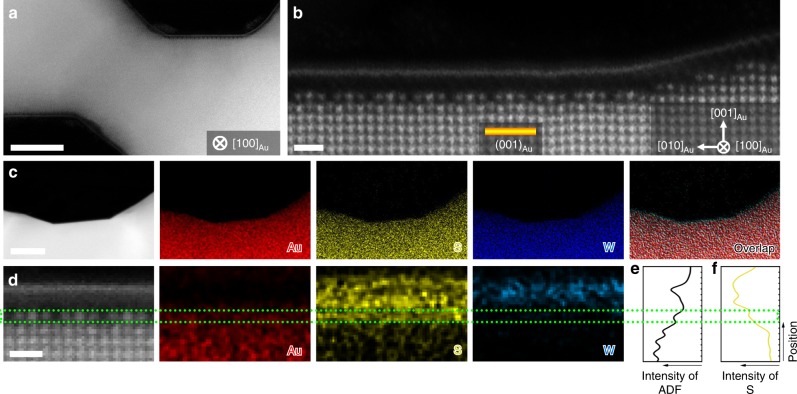
Structural and chemical characterizations of the interface between monolayer WS_2_ and NPG. **a** HAADF-STEM image of the monolayer WS_2_ grown on NPG. Scale bar, 10 nm. **b** Atomic-resolution HAADF-STEM image of monolayer WS_2_ grown along the NPG surfaces of NPG. Scale bar, 0.5 nm. **c** EDS elemental mappings and simultaneously HAADF-STEM image of WS_2_ on Au (001). Scale bar, 50 nm. **d** EELS elemental mappings and simultaneously HAADF-STEM image of monolayer WS_2_ grown on Au (001) surface. Scale bar, 0.5 nm. **e**, **f** Intensity profiles measured from the HAADF-STEM image (**e**) and S EELS mapping (**f**).

Besides MoS_2_, we also found that the interfacial reconstruction occurs in the contact between monolayer WS_2_ and NPG. As shown in Fig. 4a, the internal surface of NPG is covered by monolayer WS_2_, which is grown by the similar CVD process. The same surface reconstruction of Au (001) can be observed from the atomic-scale HAADF-STEM image viewed from [110]_Au_ direction (Fig. 4b). The direct comparison of the reconstructed Au (001) surfaces under MoS_2_ and WS_2_ is shown in Supplementary Fig. 5. The chemical distribution from energy disperse spectroscopy (EDS) elemental mappings confirms the heterostructure of monolayer WS_2_ lying on the Au surface (Fig. 4c). Moreover, the EELS mappings reveal the refined chemical information that the reconstructed interfacial phase consists of Au and S atoms (Fig. 4d). The intensity profiles measured from the HAADF-STEM image and S *L*_2,3_ EELS mapping along the out-of-plane direction are shown in Fig. 4e, f, confirming that S atoms also involve into the structural reconstruction of the Au topmost surface in the WS_2_-NPG system.

We also investigated the interfaces between monolayer TMD films and other Au surfaces with different crystallographic orientations. The interfacial reconstructions have been observed from Au {110}, {111}, {012}, and {113} surfaces. Again, sulfur can be detected from the reconstructed Au topmost surfaces by EELS spectra and elemental mappings (Supplementary Fig. 6). The atomic structures of these reconstructed interfaces appear different from that of Au {100}. The details of the structural characterization and modeling will be discussed in a separate paper.

### Structural determination of the reconstructed MoS_2_/Au {001} interface

**Fig. 5:**
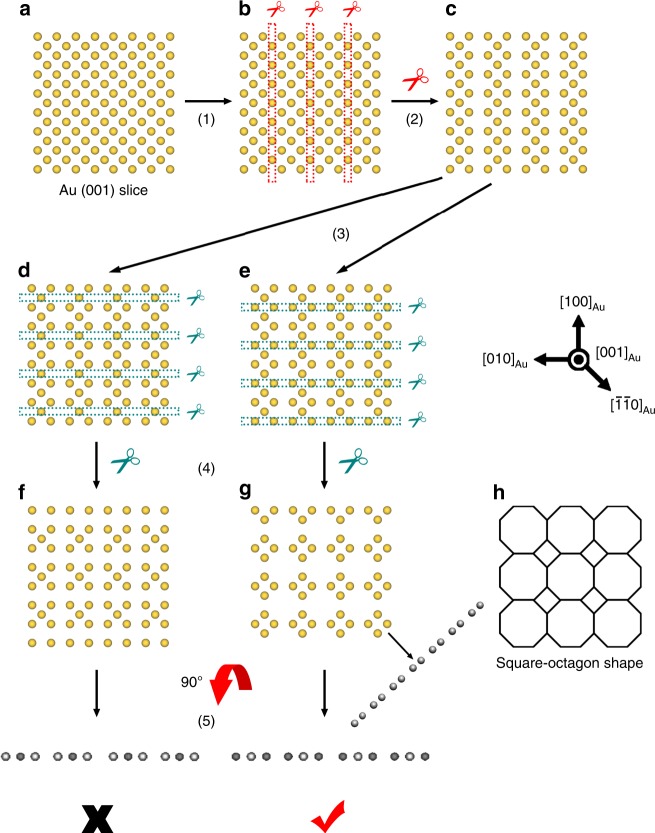
Structural determination of Au atom position in the reconstructed Au (001) surface on the basis of the HAADF-STEM images. **a** A monolayer Au (001) slice viewed from Au [001] direction. **b** Deleting the atoms in one column every three columns to make the gaps meet the periodicity viewed from Au [100] direction. **c** The structure with remaining Au atoms after process in (**b**). **d**, **e** Two different ways to delete the atoms in one row every three rows to make the gaps meet the periodicity viewed from Au [100] direction. **f** The structure with remaining Au atoms after process in (**d**), which is not consistent with the experimental HAADF-STEM images. **g** The structure with remaining Au atoms after process in (**e**), which is consistent with the experimental HAADF-STEM images in every directions we have observed. **h** The square-octagon structure composed by the remaining monolayer Au atoms.

The intensity of an atomic column in HAADF-STEM images is proportional to ~*Z*^*n*^, where *Z* denotes atomic number^43^ and *n* is a constant in the range 1.6–1.9 for most cases^44^. The contrast of S (*Z* = 16) residing at the reconstructed Au (001) surface is too low, in comparison with Au (*Z* = 79), to be identified from the HAADF-STEM images. Thus, the image contrast of the atomic columns at the Au {001} topmost surface is dominantly from Au atoms, and the relatively dark contrast represents fewer Au atoms in the columns. Based on the HAADF-STEM projections along both [110]_Au_ and [100]_Au_ directions, we can deduce that the atomic arrangements of the topmost Au atoms exhibit a square-octagon lattice from the top view of the reconstructed Au (001) surface (Fig. 5). Each vertex of the square lattice is decorated with a tilted square^45^. This structure has been predicted to be a topological phase^46^ or quantum magnetic phase^47^ by density functional theory (DFT) calculations. As to the Au (001) sublayer, it is only composed by Au atoms and the columns with weaker contrast represent the periodic absence of Au atoms (Supplementary Fig. 7b).

**Fig. 6:**
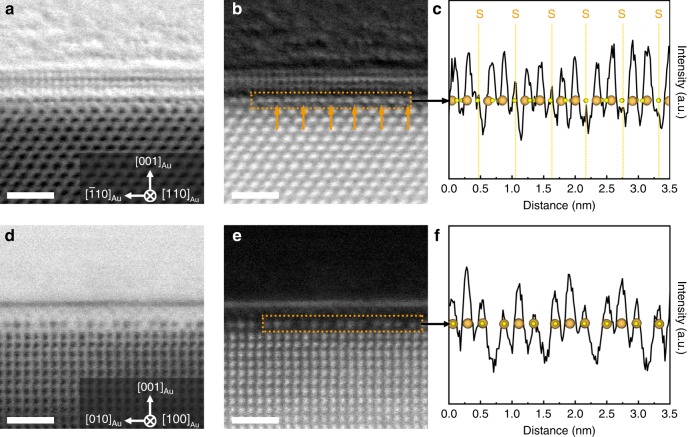
Characterization of S atom occupation in the reconstructed Au (001) surface. **a** ABF-STEM image viewed from the [110]_Au_ direction. Scale bar, 1 nm. **b** Contrast-inversed image of (**a**) showing the S atomic columns. Scale bar, 1 nm. **c** The corresponding intensity line profile of the reconstructed layer from dotted box in (**b**). **d** ABF-STEM image viewed from the [100]_Au_ direction. Scale bar, 1 nm. **e** Contrast-inversed image of (**d**) in which S atomic columns are invisible. Scale bar, 1 nm. **f** The corresponding intensity line profile of the reconstructed layer from dotted box in (**e**). The orange and yellow spheres represent Au and S atoms, respectively.

**Fig. 7:**
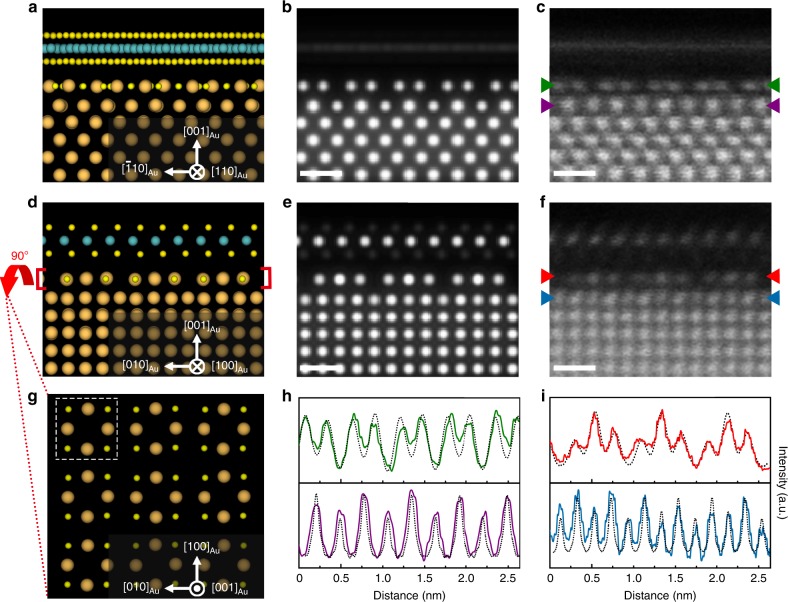
Atomic configuration of the reconstructed interface between MoS_2_ and Au (001). **a**, **d** The DFT-optimized structure model viewed from the [110]_Au_ (**a**) and [010] _Au_ (**d**) directions, respectively. **b**, **e** Simulated HAADF-STEM images from the DFT-optimized structure model viewed from the [110]_Au_ (**b**) and [010] _Au_ (**e**) directions. Scale bar, 0.5 nm. **c**, **f** Experimental HAADF-STEM images showing good match with the simulated ones viewed from the [110]_Au_ (**c**) and [010] _Au_ (**f**) directions. Scale bar, 0.5 nm. **g** The reconstructed Au (001) surface viewed from the [001] _Au_ direction (i.e., top view). The MoS_2_ monolayer and Au matrix are omitted for clarity. The dotted box shows the unit cell of an Au_4_S_4_ ring. **h**, **i** Line profiles showing the intensity variation of atomic columns viewed from the [110]_Au_ (**h**) and [010] _Au_ (**i**) directions. The green and purple lines (**h**) are detected along the Au_4_S_4_ monolayer and Au sublayer following the corresponding arrows in (**c**). The red and blue lines (**i**) are detected along the Au_4_S_4_ monolayer and Au sublayer following the corresponding arrows in (**f**). The dashed lines are detected from the same areas in corresponding simulated images. The orange, green and yellow spheres represent Au, Mo, and S atoms, respectively.

To locate the occupation of S atoms in the reconstructed Au {001} surface, we performed annular bright-field (ABF) STEM observations (Fig. 6). Additional weak contrast from S can be recognized at the gap between two pairs of Au atomic columns from the ABF-STEM image taken along the [110]_Au_ direction as well as the corresponding intensity line profile. On the contrary, no extra contrast can be detected from [100]_Au_ ABF-STEM images because S atoms overlap with Au atoms in the atomic columns along the [100]_Au_ direction. Combining the fact that the Au–S bonds are commonly realized as staple-like S–Au–S linear structures at thiolate-protected gold surfaces and interfaces^26,48^, the reconstructed Au {001} surface can be assembled by planar Au_4_S_4_ square rings shown in the dotted box of Fig. 7g, which has been observed in sulfur-containing metalloid gold clusters^49^. This surface atomic configuration gives the best match with the HAADF- and ABF-STEM images in both [110]_Au_ and [100]_Au_ directions, as well as the chemical analyses from XPS and EELS.

To verify and optimize the reconstructed surface structure of Au {001} derived from the STEM observations, we conducted DFT calculations (see the Methods section). The primary structural models were based on the HAADF-STEM images with the orientation relationship between Au and monolayer MoS_2_ of (001)_Au_ // (001)_MoS2_ and [100]_Au_ // [010]_MoS2_, which is commonly observed in our study. After relaxation, the lattice mismatch is only 1.77% between monolayer MoS_2_ and Au_4_S_4_. The DFT-optimized structure viewed from [110]_Au_ and [010] _Au_ directions are shown in Fig. 7a, d, respectively. The simulated HAADF images (Fig. 7b, e) from corresponding directions of the structure model are in remarkable consistency with the experimental ones (Fig. 7c, f). Quantitative image intensity analyses (Fig. 7h, i) of the Au_4_S_4_ monolayers and Au sublayers from the experimental (solid lines with different color) and simulated images (dashed lines) also shows good agreement from both projection directions. Moreover, the distance of 2.67 Å between the Au_4_S_4_ monolayer and the bottom S layer of MoS_2_ is also close to the experimental measurement (~2.56 Å). Figure 7g exhibits the top view of the reconstructed Au (001) surface with the formation of Au_4_S_4_ ring motifs. Its orientation relations with the Au matrix, sublayer, and MoS_2_ layer are shown in Supplementary Fig. 7.

### Factors influencing the interfacial Au_4_S_4_ phase formation

**Fig. 8:**
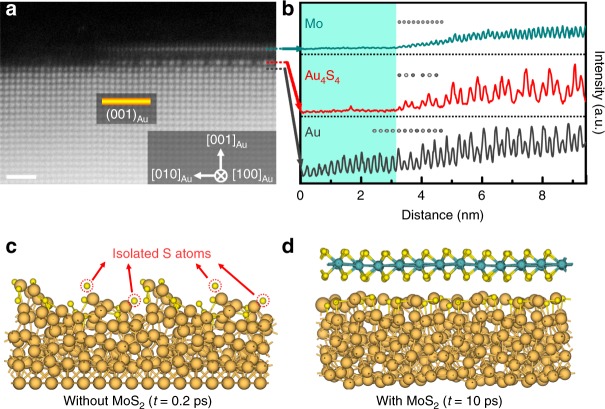
The co-existence relationship between MoS_2_ and reconstructed Au_4_S_4_ structure. **a** HAADF-STEM image of a partially covered Au (001) surface by monolayer MoS_2_ viewed from the [100]_Au_ direction. Scale bar, 1 nm. **b** The corresponding intensity line profile of Mo layer in MoS_2_ (green line), Au_4_S_4_ interface layer (red line) and Au sublayer (gray line). **c**, **d** Stability of the Au_4_S_4_ phase in MD simulations without (**c**) or with (**d**) a monolayer MoS_2_ top. The orange, green and yellow spheres represent Au, Mo, and S atoms, respectively.

There is a strict co-existence relation between the reconstructed Au (001) surfaces and 2D TMDs. The surface reconstruction only takes place at the 2D TMD/Au interfaces. We intentionally shortened the CVD growth time of MoS_2_ from 20 min to 10 min to make NPG internal surfaces partially covered by monolayer MoS_2_. Figure 8a shows the Au (001) surface on which the Au_4_S_4_ reconstruction is only visible in the region covered by the MoS_2_ monolayer. With the absence of MoS_2_ layer in the left side of the (001) surface, the Au_4_S_4_ reconstruction layer also disappears. The intensity line profiles shown in Fig. 8b further verify the exact correspondence between the reconstructed Au (001) surface and 2D MoS_2_ as the Au_4_S_4_ structure disappears immediately at the edge of the MoS_2_ monolayer. Meanwhile, the contrast variation of the Au (001) sublayer also disappears together with the absence of the reconstructed topmost surface. These structural features unambiguously demonstrate that the Au_4_S_4_ reconstruction is stimulated and stabilized by MoS_2_ and the sublayer reconstruction, most likely as the buffer layer, correlates with the topmost Au_4_S_4_ reconstruction. Importantly, in comparison with the unreconstructed region of the (001) surface, the Au_4_S_4_ reconstruction is one atomic layer high and forms an atomic step at the site between reconstructed and unreconstructed (001) surface. It provides compelling evidence that the Au atoms in Au_4_S_4_ monolayer most likely come from the sublayer and are “dragged” out by S atoms to form the two-atomic-layer interfacial phase. Similar phenomena can also be observed from other Au facets, such as {110} and {113} (Supplementary Fig. 8). We also carefully characterized the surface structure of the NPG sample, which is annealed in sulfur-rich environment but without the Mo source for MoS_2_ growth at the CVD temperature of 873 K. Again, no reconstructed surfaces can be seen except that some sulfur particles attach on Au surfaces (Supplementary Fig. 9). We performed molecular dynamics (MD) simulations on the thermal stability of the reconstructed Au (001) surface with or without MoS_2_ coverage (for details, see the Methods section). The reconstructed surface without MoS_2_ coverage disappears shortly after 0.2 ps by decomposing as elemental S atoms and Au (Fig. 8c). In contrast, when covered by MoS_2_, the reconstructed Au_4_S_4_ structure can retain well at 900 K in the maximum simulation time period of 10 ps in this study (Fig. 8d). The enhanced thermal stability of the interfacial phase is apparently related to the vdW interaction with the top MoS_2_ monolayers although further investigation is required^50^.

We also investigated the effects of excess sulfur, CVD temperatures and Mo sources on the interfacial reconstructions. After largely decreasing the loading amount of sulfur powders from 1 g to 200 mg for CVD growth, unreconstructed interfaces between MoS_2_ and Au can be observed (Supplementary Fig. 10), suggesting that the S-rich environment is necessary for the formation of the interfacial Au_4_S_4_ phase. In contrast, when we changed the Mo source from MoO_3_ to MoCl_5_ and reduced the CVD temperature from 923 to 723 K, the interfacial reconstruction is not affected and always occurs accompanying with the formation of MoS_2_ (Supplementary Fig. 11).

## Discussion

**Fig. 9:**
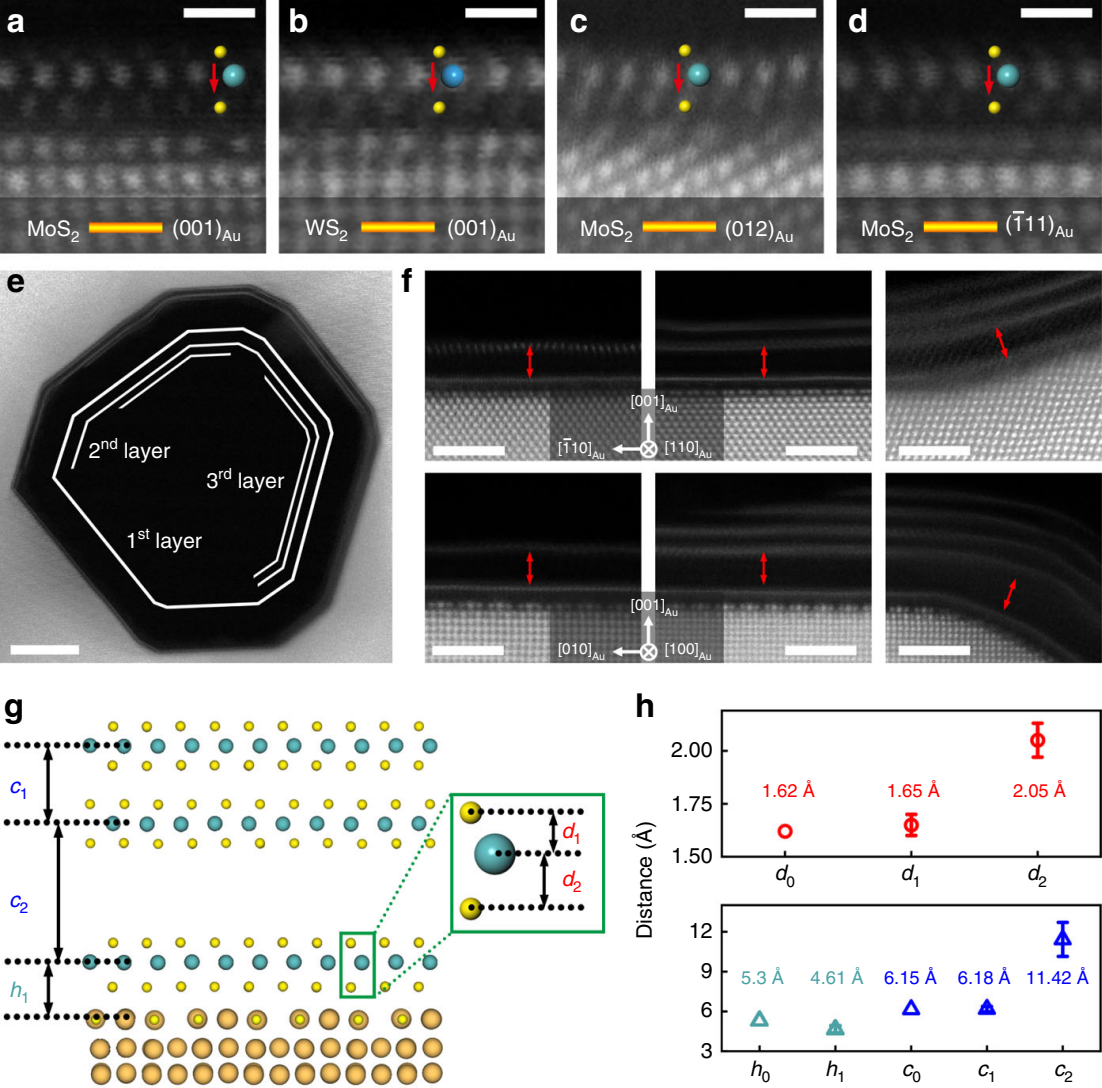
Structure of monolayer and multi-layer MoS_2_ on reconstructed Au (001) surfaces. **a**–**d** HAADF-STEM imaging showing the cross-sectional structures of MoS_2_ and WS_2_ monolayers on the reconstructed Au (001) surfaces. Scale bar, 0.5 nm. **e** Multi-layer MoS_2_ on the internal surface of NPG. Scale bar, 5 nm. **f** Atomic-resolution HAADF-STEM images of multi-layer MoS_2_ grown on Au surfaces viewed from the [100]_Au_ and [110]_Au_ directions. Scale bar, 2 nm. **g** Structure model of the MoS_2_ on the reconstructed Au (001) surface. The *d* is defined as the distance between Mo atom layer and S atom layer, *c* is the interlayer spacing between two MoS_2_ layers and *h* is the distance between topmost Au atom layer and the nearest S atom layer in the top MoS_2_. **h** The comparison of *d*, *h*, and *c* with the values of *d*_0_, *h*_0_ (ref. ^13^), and *c*_0_ in pristine MoS_2_. The error bars represent the standard deviation of each distance. Source data are provided as a Source Data file. The orange, green, blue and yellow spheres represent Au, Mo, W, and S atoms, respectively.

We noticed that the sulfur-induced gold surface reconstruction, in turn, alters the structure of 2D TMDs. It was found that the reconstructed Au surface leads to the inverse symmetry breaking of the S-Mo-S in top 1H MoS_2_. The distances between Mo layer and two S layers, which should be the same as *d*_0_ ~1.62 Å, change to two different values (*d*_1_ and *d*_2_). The one that is close to the reconstructed interface is 2.05 Å ± 0.08 Å (*d*_2_ in Fig. 9h), which is obviously larger than *d*_*1*_ that remains to be close to *d*_0_ (*d*_1_, ~1.65 Å) (Fig. 9a–d). In addition to the asymmetric S-Mo-S structure of 1H MoS_2_, the distance between the reconstructed Au surface and the Mo layers also becomes smaller (4.65 Å ± 0.27 Å, *h*_1_ in Fig. 9h) in comparison with that of the unreconstructed interface (*h*_0_, ~5.30 Å)^13^. Furthermore, the interlayer spacing between the first two MoS_2_ layers was significantly expanded when multi-layer MoS_2_ was grown on NPG by increasing the deposition time (Fig. 9e). The magnified HAADF-STEM image (Fig. 9f) clearly shows the enlarged interlayer spacing of 11.42 Å ± 1.51 Å (c_2_ in Fig. 9h), which is almost twice larger than the normal one (6.15 Å). Although the vertical distance between the bottom S layer and the topmost Au_4_S_4_ is ~2.56 Å, the minimum interatomic distance between a sulfur atom in the bottom S layer of MoS_2_ and a gold atom in the topmost Au_4_S_4_ layer is 3.04 Å (Supplementary Fig. 12b), which is much larger than the covalent Au–S bonding lengths (~2.42 Å)^8^. Therefore, the interaction between monolayer MoS_2_ and Au_4_S_4_ reconstructed surface should still be the vdW type. This assumption is supported by DFT calculations. In the isosurface of partial charge density, there is no charge redistribution and superposition at the interface between MoS_2_ and reconstructed Au_4_S_4_ (Supplementary Fig. 12c).

**Fig. 10:**
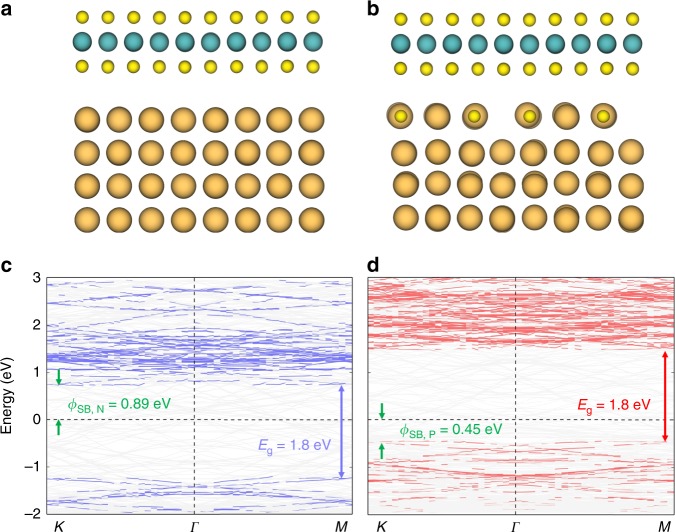
The band structures of MoS_2_–Au (001) interface without and with Au_4_S_4_ reconstruction. **a**, **c** The side view of the model of unreconstructed MoS_2_–Au (001) interface (**a**) and the corresponding band structure (**c**). **b**, **d** The side view of the model of reconstructed MoS_2_–Au_4_S_4_ interface (**b**) and the corresponding band structure (**d**). The orange, green and yellow spheres represent Au, Mo, and S atoms, respectively. The zero of energy is set at the Fermi level. *E*_g_ denotes the band gap of monolayer MoS_2_, and *Φ*_SB, N_ (*Φ*_SB, P_) denotes n-type (p-type) SBH for electrons (holes).

Since structural changes often result in new functionalities of materials, the interfacial reconstruction between MoS_2_ and Au may open a way to tailor the properties of 2D TMDs and their devices. Our preliminary DFT study (Fig. 10) suggests that the formation of Au_4_S_4_ interfacial phase leads to the transition of the original n-type vdW contact between MoS_2_ monolayer and Au (001) electrode to a p-type contact with the reduced Schottky barrier height from 0.89 eV (*Φ*_SB,N_ = *E*_CB_ − *E*_f_) to 0.45 eV (*Φ*_SB,_ P = *E*_VB_ − *E*_f_). This transition may be utilized to realize the n/p-type conversion in 2D TMD devices and the lower energy barrier may benefit the hole mobility in p-type transistors^51,52^. While, the DFT calculations show that the vertically distorted MoS_2_ monolayer on the reconstructed Au_4_S_4_ is not energetically stable and becomes to be more symmetric during relaxation. The divergence between our experimental observations and DFT calculations could be caused by the complexity of the real interfacial structure. Although the vertical distortion alone can obviously affect the bandgaps of MoS_2_ (Supplementary Fig. 13), it may not significantly alter the vdW contact between MoS_2_ monolayer and Au (001) surface in which the reconstructed Au_4_S_4_ interfacial phase plays a more important role in the transition from n-type to p-type vdW contacts.

In conclusion, we observed the interfacial reconstruction at Au (001)/monolayer TMDs junctions. The reconstructed Au_4_S_4_ structure co-exists with and is stabilized by the top TMD monolayers with an enhanced vdW interaction. The unique vdW contact between TMDs and Au by interfacial reconstruction may pave a new way to fabricate perfect vdW interface with tunable the Schottky barrier height and contact types and to develop new functionalities of TMDs-metal heterostructures.

## Methods

### Preparation of NPG substrates

The NPG substrates were prepared by chemically dealloying Ag_65_Au_35_ (atomic percentage) films with a thickness of 100 nm in a 70 vol% HNO_3_ solution (Aldrich) for 3 h at room temperature^33^, by which silver was selectively dissolved from the alloy and left behind an NPG membrane. After removing the residual chemicals by rinsing, the NPG films were placed on a silicon wafer with 200 nm thick SiO_2_ as the 3D porous substrates for CVD growth of MoS_2_ or WS_2_.

### CVD growth of TMDs on NPG

The MoS_2_ or WS_2_ monolayer and multilayer films were grown on NPG using a three-temperature-zone CVD system. As shown in Supplementary Fig. 1, ~1 g sulfur (Aldrich, purity 99.9%), ~0.02 g MoO_3_ powders (Aldrich, purity 99.9%) and the substrate (100 nm thickness NPG on oxidized silicon) were placed from upstream to downstream at the three zones with different temperatures, respectively. Ultra-pure argon was used as the carrier gas and the pressure inside the tube was controlled by adjusting the Ar flow rate. During the reaction process, the Ar flow rate was 600 sccm, and the temperatures of three zones were 120 °C, 550 °C and 650 °C, respectively. After the reaction time of 20 min, MoS_2_ monolayers completely covered the internal surfaces of NPG. Multilayer MoS_2_ films were obtained by extending the reaction time to 30 min. As shown in Supplementary Fig. 1c, the synthesis of WS_2_ films was similar to that of MoS_2_. ~0.02 g WCl_6_ powders (Aldrich, purity 99.9%) and ~1 g sulfur (Aldrich, purity 99.9%) were placed from upstream to downstream respectively as precursor sources, and the heating temperature were 55 °C and 120 °C during the CVD process.

### STEM Characterization

The microstructures and atomic structures of MoS_2_ and WS_2_ on NPG were characterized by a transmission electron microscope (JEOL ARM 200F) equipped with a cold emission gun and an aberration corrector for the probe-forming lens system. The collection angle of the HAADF detector ranges from 81 to 228 mrad, and the collection angle of the ABF detector ranges from 6 to 25 mrad. The filtered STEM-HAADF images with temperature color in Figs. 1 and 3 were deconvoluted with *HREM DeConvHAADF* software (HREM Research Inc.)^53^. The rest images used in the paper are all original ones.

### EELS Characterization

The EELS analyses were performed with the *Gatan GIF Quantum* system on STEM mode. The EELS elemental mapping was simultaneously recorded with HAADF-STEM. The pixel size is 0.32 Å × 0.32 Å and a short dwell time of 0.01 s/pixel was used in order to avoid the spatial drift and irradiation damage. S *L*_2,3_ edges presented in Fig. 1e are the integrated signals from all the pixels in 3 rows for each defined layer (Supplementary Fig. 14). The width of 3 rows (0.96 Å) is smaller than the atomic radius (S, 1.09 Å; Mo, 1.36 Å; Au, 1.44 Å). There is a gap spacing between two adjacent layers with more than 4 rows (1.28 Å) to avoid possible interference from scanning drifts. In this way, we got the original EELS spectra of these six layers (Supplementary Fig. 15). To better show the characteristic EELS edges and peaks, we smoothed these spectra with low-pass numerical filtering. The background of sulfur core-loss edges was subtracted using a power law fitting. All the smoothing and subtracting processes are performed using the *Digital Micrograph* software package (Gatan Inc.).

### STEM image simulation

The simulations of HAADF-STEM images in Fig. 7 were conducted by *xHREM* software with *STEM Extension* (HREM Research Inc.)^54^, and the absorption of thermal diffuse scattering was taken into account for each element. The defocus value of ~5 nm was adopted and the specimen thickness of ~5 nm was used. The 200 kV probe with a probe-forming aperture of 22 mrad was postulated to be aberration-free. The collection angle of the HAADF detector is set from 81 to 228 mrad.

### DFT calculations

Density functional theory based First-principles calculations were carried out using FHI-aims code^55,56^, which is an all electron, full potential electronic structure code by using a numeric, atom-centered basis set. The electronic exchange and correlation were treated with the generalized gradient approximation (GGA) of Perdew–Burke–Ernzerhof (PBE)^57^. All the numeric settings were chosen as an energy convergence less than 10^−3^ eV is achieved. The atomic positions were allowed to fully relax using the Broyden–Fletcher–Goldfarb–Shanno (BFGS) algorithm. Using DFT implemented CP2K package^58,59^, ab initio molecular dynamics simulations for the reconstructed and unreconstructed interface were performed in the canonical ensemble by using the Nose-Hoover thermostat and a time step of 1 fs at a finite MD temperature of 900 K. The exchange correlation potential was described by the GGA with the spin-polarized functional of PBE^57^. Wavefunctions were expanded in triple-ζ Gaussian basis sets with an auxiliary plane-wave basis and a cutoff energy of 300 Ry. Core electrons were modeled by scalar relativistic norm-conserving pseudopotentials^60,61^.

## Supplementary information

Supplementary Information

## Data Availability

The source data underlying Fig. 9h are provided as a Source Data file. Other data that support the findings of this study are available from the corresponding authors upon request.

## Source data

Source Data
