# Supplementary material for: Van der Waals interfacial reconstruction in monolayer transition-metal dichalcogenides and gold heterojunctions
Source: Nat Commun. 2020 Feb 21;11:1011. doi: 10.1038/s41467-020-14753-8 (PMC7035323; doi:10.1038/s41467-020-14753-8)
Supplement: Supplementary file 1 — Supplementary Information [file 41467_2020_14753_MOESM1_ESM.pdf]

Supplementary Materials for

Van der Waals interfacial reconstruction in monolayer transition-metal dichalcogenides and gold heterojunctions

Luo et al.

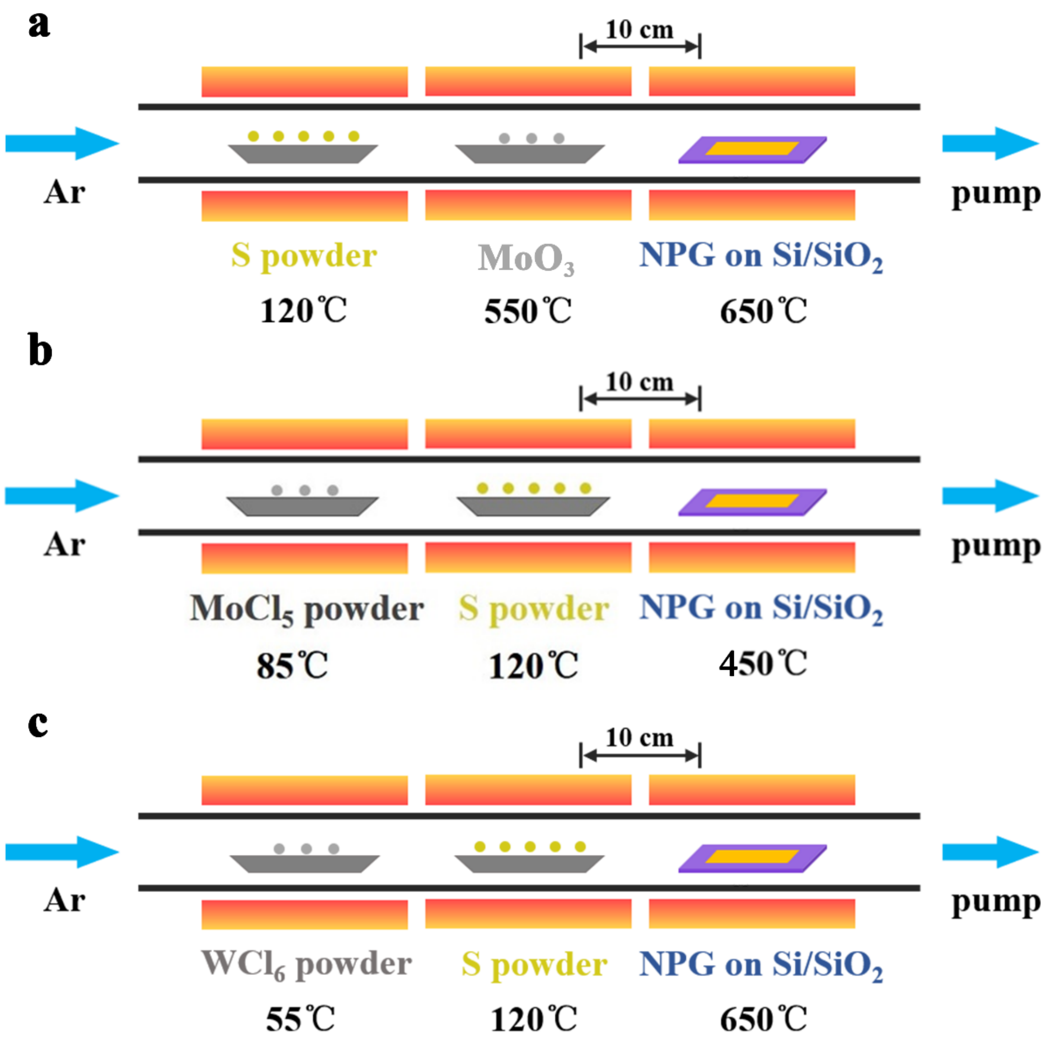

Supplementary Figure 1 | Schematics of the CVD growth of TMDs on NPG under different conditions. a, Using MoO_3 as Mo source to grow MoS_2 on NPG at 650°C (923 K). **b,** Using $MoCl_5$ as Mo source to grow MoS_2 on NPG at 450°C (723 K). **c,** Using WCl_6 as W source to grow WS_2 on NPG at 650°C (923 K).

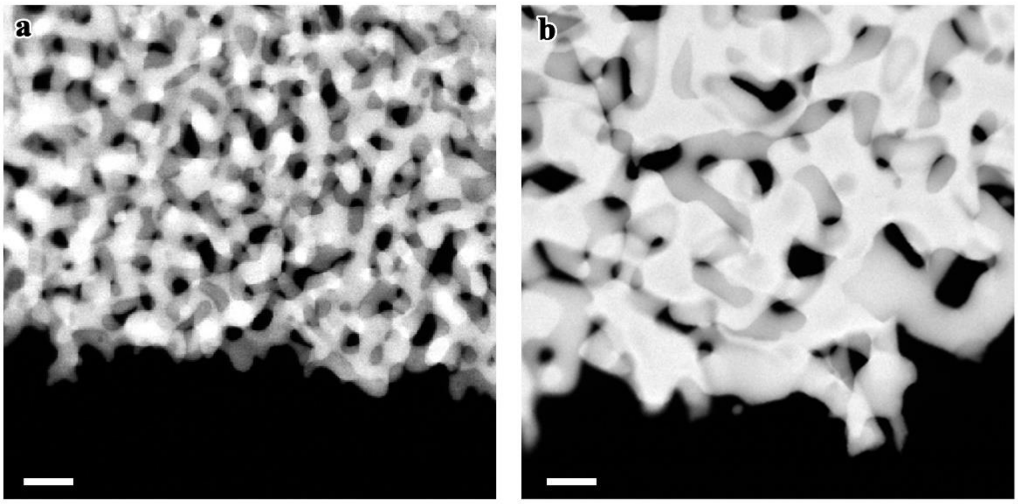

Supplementary Figure 2 | Microstructure of dealloyed NPG films before and after CVD. a, The as-prepared NPG with an average pore size ~ 20 nm. **b,** The NPG with an average pore size ~ 50 nm after CVD growth of MoS_2. Scale bar, 50 nm.

Supplementary Table 1 | XPS quantitative analysis of MoS_2 grown on glass and NPG.

	Mo (atom %)	S (atom %)	Mo/S
MoS_2 on glass (1)	2.84	5.36	0.53
MoS_2 on glass (2)	2.68	4.94	0.54
MoS_2 on glass (3)	1.84	3.46	0.53
MoS_2 on NPG (1)	0.74	1.64	0.45
MoS_2 on NPG (2)	1.45	3.05	0.48
MoS_2 on NPG (3)	3.06	7.50	0.41

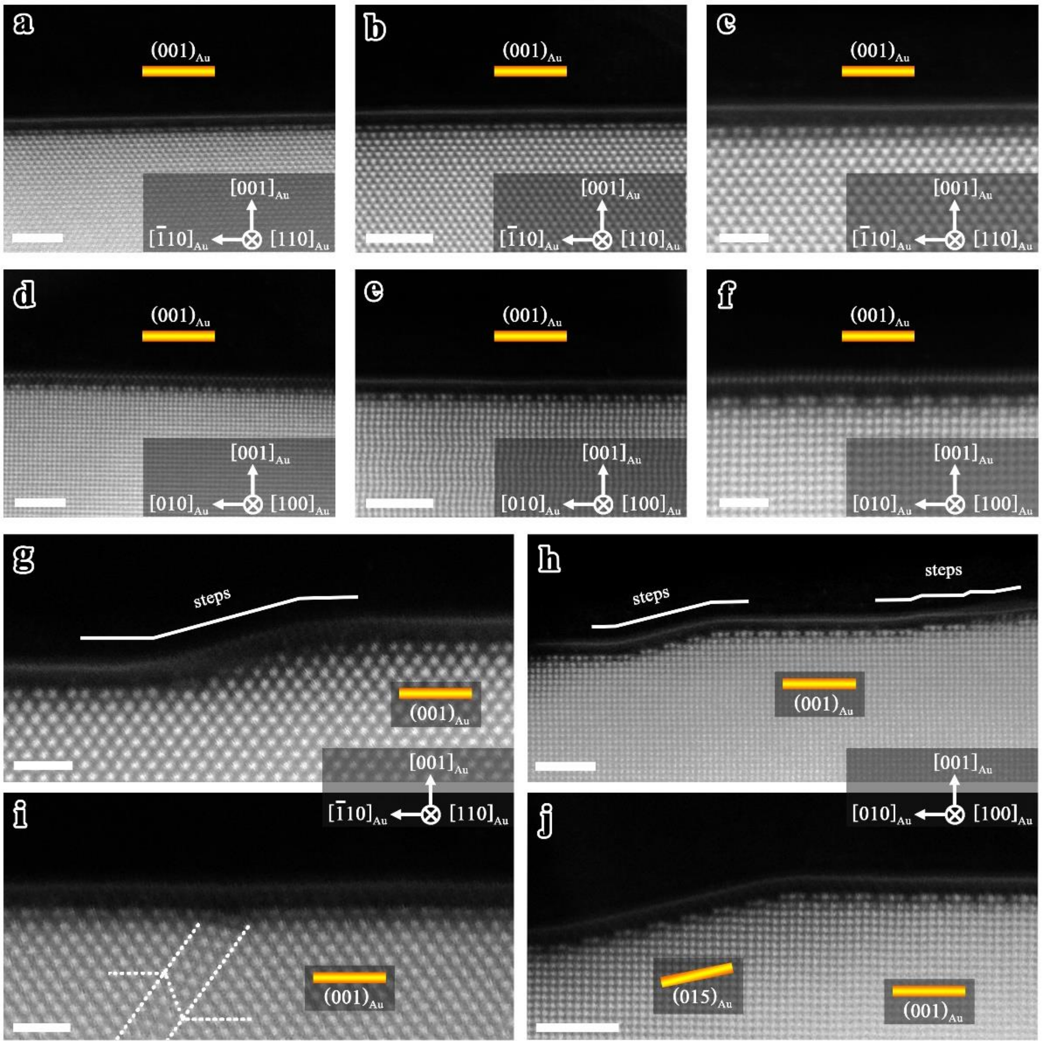

Supplementary Figure 3 | Structural characterization of the interfaces between monolayer MoS_2 and Au (001) surfaces. a-c, HAADF-STEM images viewed from the [110] $_{Au}$ direction. **d-f,** HAADF-STEM images viewed from the [100] $_{Au}$ direction. **g, h,** HAADF-STEM images of monolayer MoS_2 growing across the steps on Au (001) surfaces. **i,** HAADF-STEM image of monolayer MoS_2 growing across a twin step on Au (001) surface. **J,** HAADF-STEM image of monolayer MoS_2 growing continuously on Au (001) and (015) surface. Scale bar, 2 nm in (**a**, **b**, **d**, **e**, **h** and **j**) and 1 nm in (**c**, **f**, **g** and **i**).

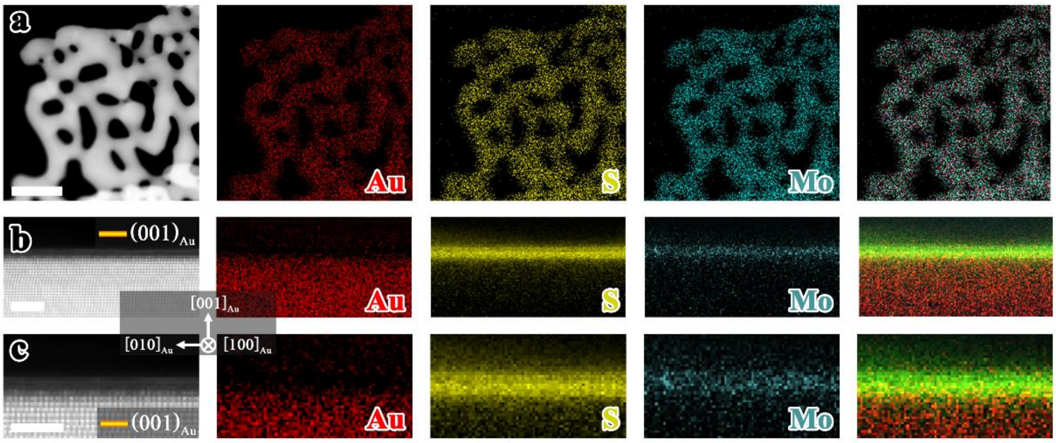

Supplementary Figure 4 | Chemical characterization of the interface between monolayer MoS_2 and Au (001) surface. a, STEM-EDS elemental mappings and HAADF-STEM image of MoS_2 growing on NPG. Scale bar, 100 nm. **b,** EELS elemental mappings and simultaneous HAADF-STEM image of MoS_2 growing on Au (001) surface. Scale bar, 2 nm. **c,** Magnified EELS elemental mappings and simultaneous HAADF-STEM image of MoS_2 growing on Au (001) surface. Scale bar, 2 nm**.**

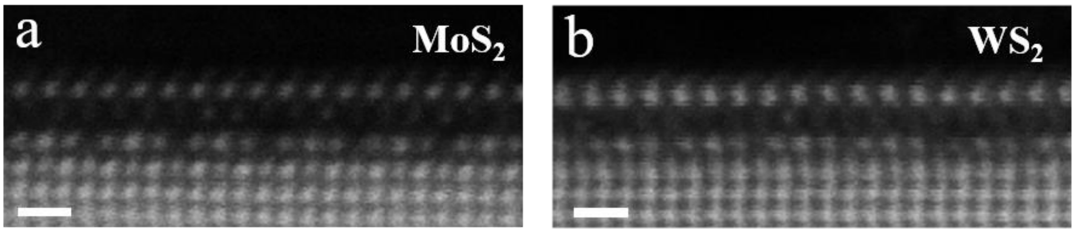

Supplementary Figure 5 | Comparison of the HAADF-STEM images of MoS_2 (**a**) and WS_2 (**b**) grown on Au (001) surfaces of NPG.

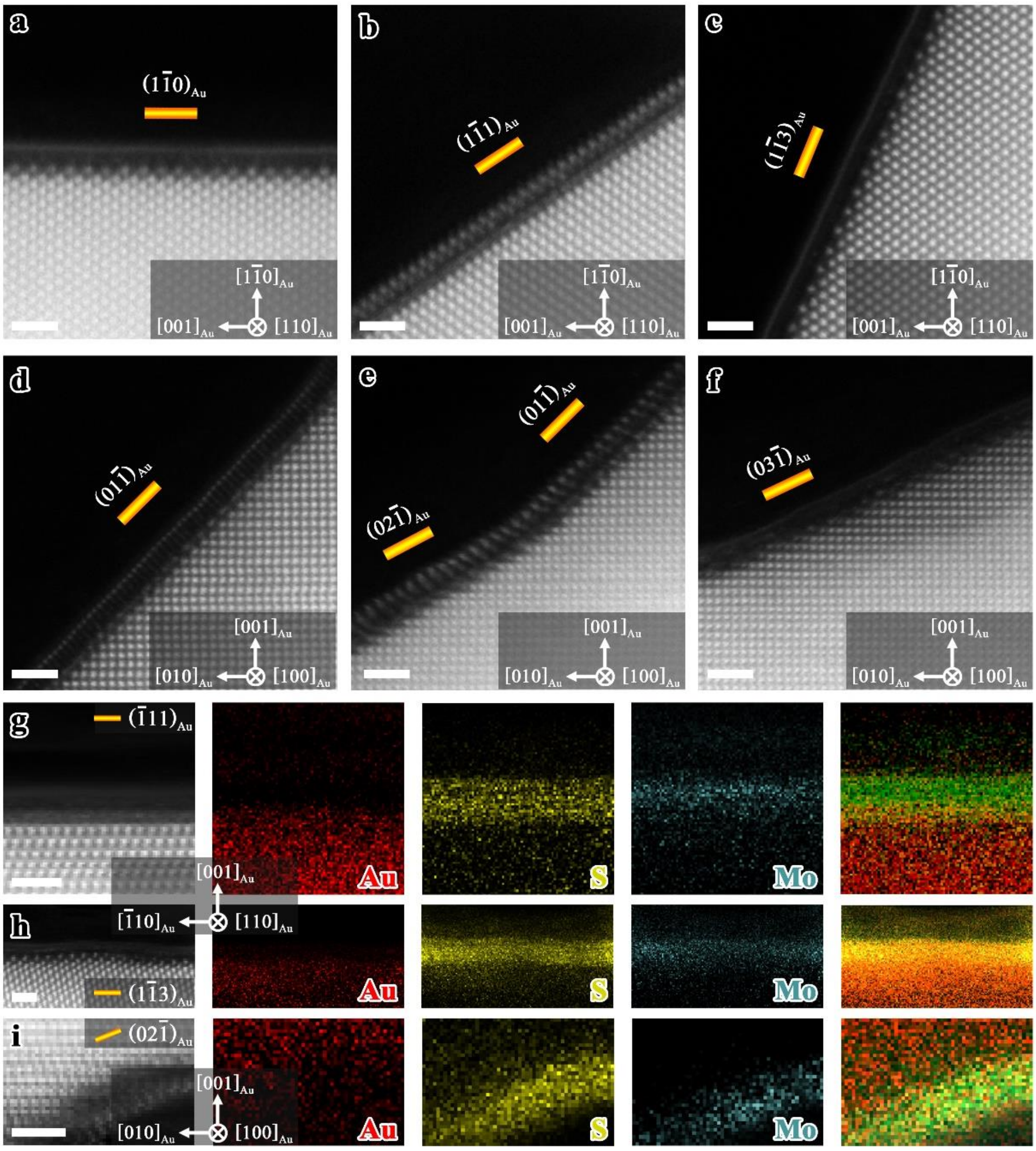

Supplementary Figure 6 | Interfaces of MoS_2 monolayers with {110}, {120} and {130} and {113} facets of NPG. a-c, HAADF-STEM images of MoS_2 monolayers growing on Au $(1\bar{1}0)$ (a), $(1\bar{1}1)$ (b) and $(1\bar{1}3)$ (c) viewed from the $[110]_{Au}$ direction. Scale bar, 1 nm. **d-f,** HAADF-STEM images of MoS_2 monolayers growing on Au $(01\bar{1})$ (d), $(02\bar{1})$ (e) and $(03\bar{1})$ (f) viewed from the $[100]_{Au}$ direction. Scale bar, 1 nm. **g-i,** EELS elemental mappings and simultaneous HAADF-STEM images of MoS_2 growing on Au $(\bar{1}11)$ (g), $(1\bar{1}3)$ (h) and $(02\bar{1})$ (i) surface. Scale bar, 1 nm.

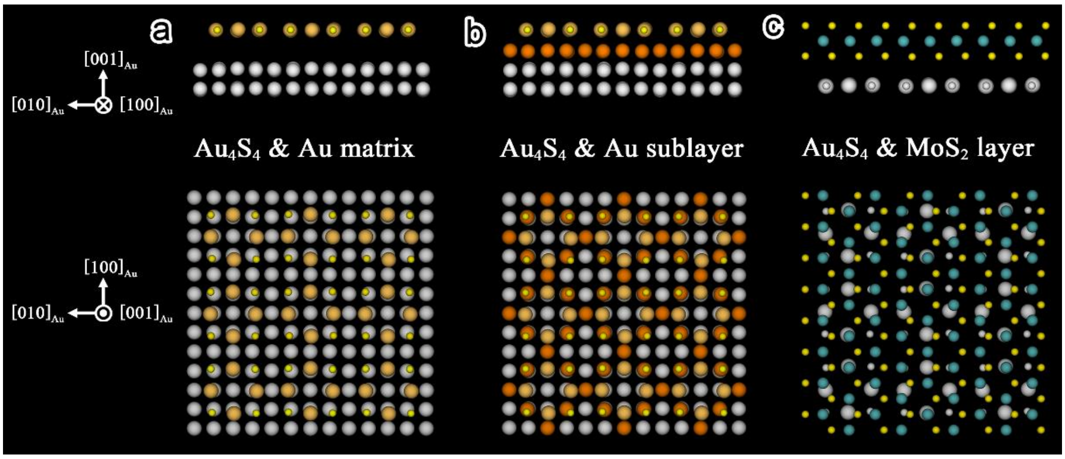

Supplementary Figure 7 | Configuration and orientation relationship of reconstructed Au_4S_4 layer with Au matrix and MoS_2 layer. a, The top and side views of Au_4S_4 reconstructed layer on Au matrix without the sublayer. **b,** The top and side views of Au_4S_4 reconstructed layer on Au sublayer with the Au matrix. **c,** The top and side views of MoS_2 monolayer on Au_4S_4 reconstructed layer. The dark yellow, orange, grey, blue and bright yellow spheres represent topmost Au atoms, sublayer Au atoms, bulk Au atoms, Mo atoms and S atoms, respectively.

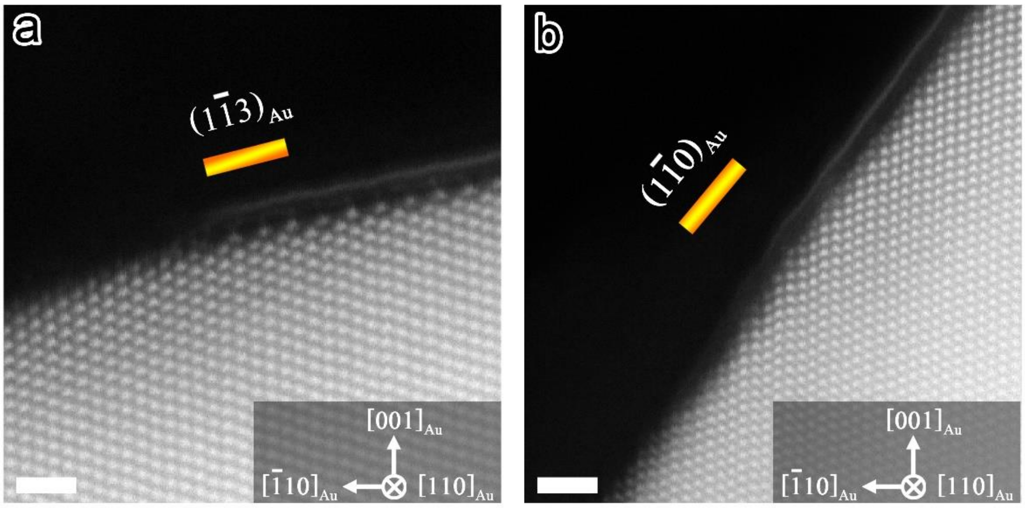

Supplementary Figure 8 | Co-existence relationship between MoS_2 and the reconstructed Au surface. a, HAADF-STEM image of a half-covered Au (1$\bar{1}$3) surface of NPG by MoS_2 viewed from the [110] $_{Au}$ direction. Scale bar, 1 nm. **b,** HAADF-STEM image of a half-covered Au (1$\bar{1}$0) surface of NPG by MoS_2 viewed from the [110] $_{Au}$ direction. Scale bar, 1 nm.

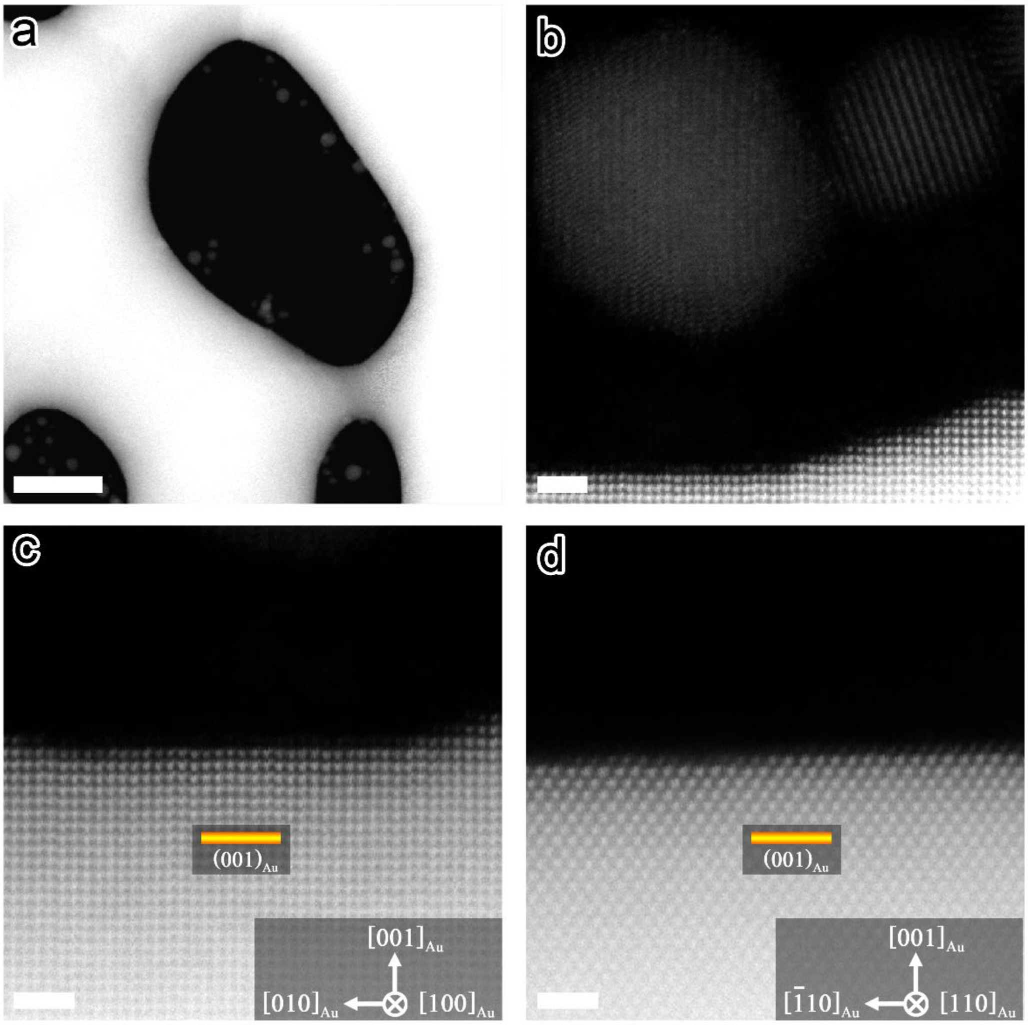

Supplementary Figure 9 | Characterization of NPG surfaces annealed in sulfur-rich environment without the deposition of MoS_2. a, Low-mag HAADF-STEM image showing S nanoparticles on internal surface of NPG. Scale bar, 20 nm. **b,** Magnified HAADF-STEM image showing the gold surface and nanoparticles. Scale bar, 1 nm. **c, d,** Atomic-resolution HAADF-STEM images viewed from the [100] Au (**c**) and [110] Au (**d**) directions and showing no reconstructions. Scale bar, 1 nm.

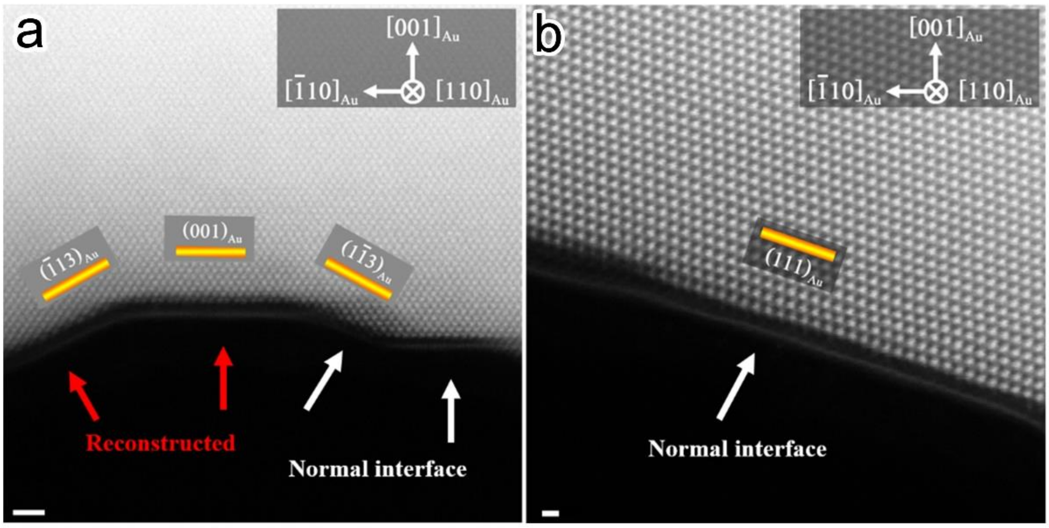

Supplementary Figure 10 | Characterization of monolayer MoS_2 grown on NPG surface with a low loading amount of S source. a, The reconstructed and unreconstructed (normal) interfaces between monolayer MoS_2 and Au surface viewed from Au [110] direction. **b,** The normal interface between monolayer MoS_2 and Au (111) surface viewed from Au [110] direction. Scale bar, 1 nm.

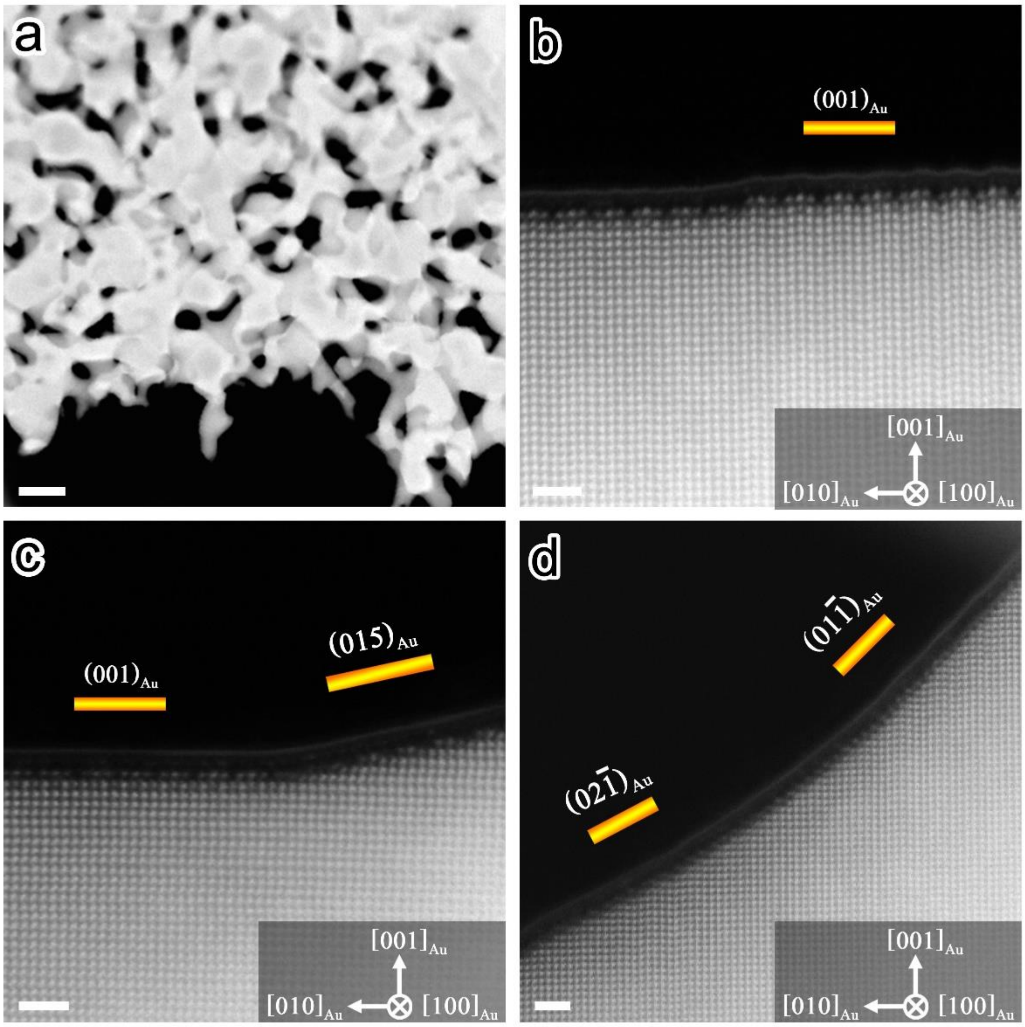

Supplementary Figure 11 | Characterization of monolayer MoS_2 grown on NPG surface at the lower temperature of 723 K. a, The NPG with the pore size ranging from 20-50 nm after CVD growth of MoS_2 at 723 K. Scale bar, 50 nm. **b-d,** Atomic-scale HAADF-STEM images of monolayer MoS_2 grown on different facets of NPG viewed from the [100] $_{Au}$ direction. Interfacial reconstruction can be observed in the sample. Scale bar, 1 nm.

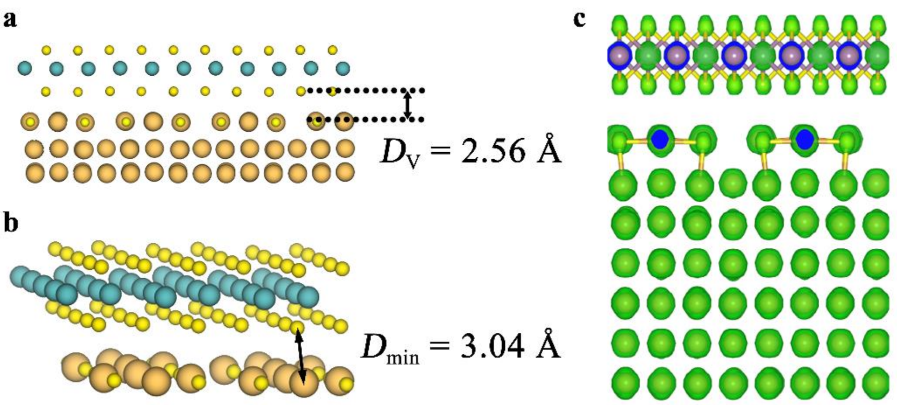

Supplementary Figure 12 | a, The vertical distance D_V straight down from the bottom S layer in MoS_2 to the Au_4S_4 layer. **b,** the minimum distance D_{min} from a sulfur atom in MoS_2 to a gold atom in Au. **c,** Calculated isosurface of the electronic charge density at the MoS_2-Au_4S_4 interface.

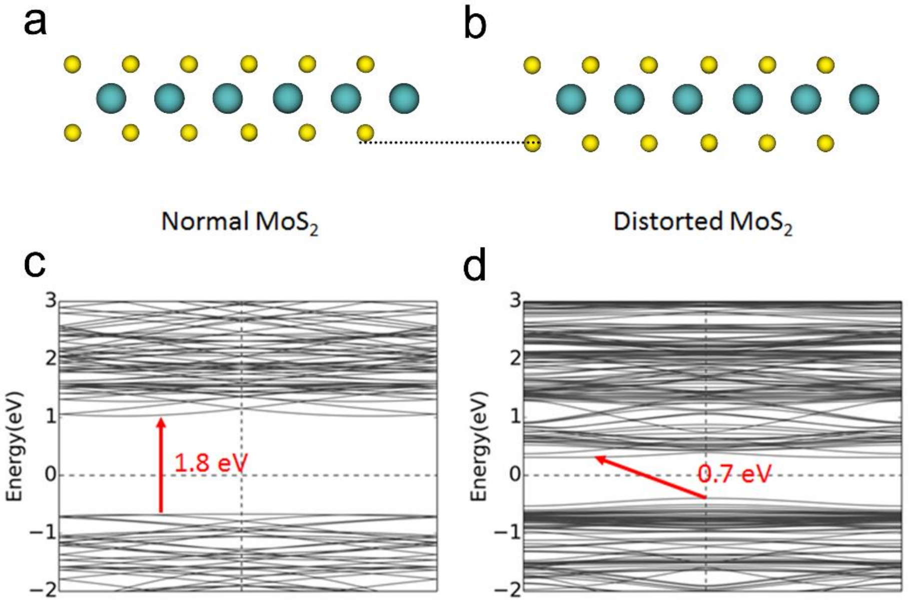

Supplementary Figure 13 | DFT calculations of the band structure of the vertically distorted MoS_2 monolayer by offsetting the bottom S layer 0.44 Å downward according to our HAADF-STEM observations. The side view of the model of monolayer MoS_2 (**a**) and the corresponding band structure (**c**). The side view of the model of monolayer MoS_2 with a distorted S layer (**b**) and the corresponding band

structure (**d**). In the calculation, the reconstructed Au_4S_4 substrate was not considered because the DFT calculations show that the vertically distorted MoS_2 monolayer on the reconstructed Au_4S_4 is not energetically stable and becomes to be more symmetric during relaxation. The divergence between our experimental observations and DFT calculations could come from the complexity of real interfacial structure or some missed factors in the calculations.

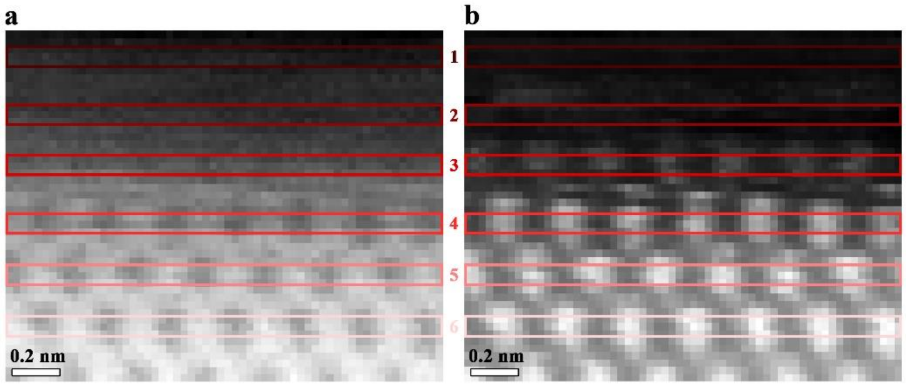

Supplementary Figure 14 | EELS spectrum image (**a**) and simultaneously gained HAADF-STEM image (**b**) with different red frames showing the pixels used to obtain the corresponding spectra in **Fig. 1e**.

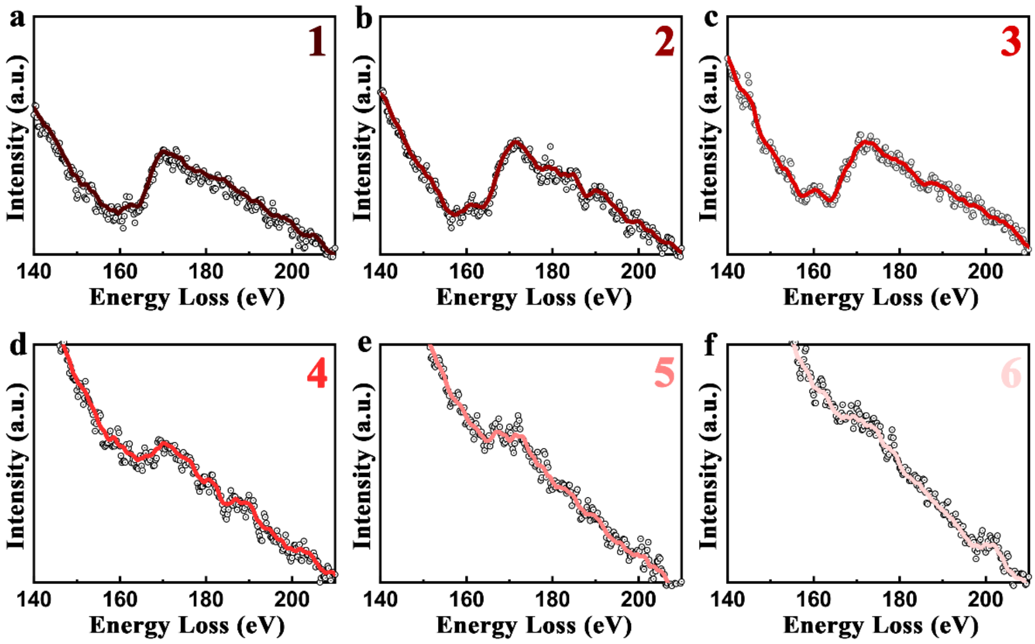

Supplementary Figure 15 | The original (black circles) and smoothed (red curves) EELS spectra from Layer #1 (**a**) to Layer #6 (**f**).
